# ‘I stand corrected’

**DOI:** 10.1259/bjrcr.20150237

**Published:** 2015-08-13

**Authors:** Sumer N Shikhare, Poh Lye Paul See, Niraj Dubey

**Affiliations:** Department of Diagnostic RadiologyKhoo Teck Puat HospitalAlexandra HealthSingapore

## Abstract

Fibular hemimelia is a rare congenital disorder with partial or complete absence of the fibula. It is usually associated with other osseous and soft tissue abnormalities of the knee joint and lower limb. Here we report an interesting case of fibular hemimelia diagnosed incidentally with characteristic radiographic and magnetic resonance imaging MRI findings in the knee joint and lower limb. The radiologists need to be aware of the typical features and associations of fibular hemimelia so as to be able to guide further orthopaedic management in these patients.

## Summary

Fibular hemimelia is a rare congenital condition with an estimated incidence between 7.4 and 20 cases per million live births. The primary feature of this condition is the partial or complete absence of the fibula. It is the most common long bone deficiency followed by defects of the tibia, ulna, radius and femur in that order.^1,2^ Fibular hemimelia is usually unilateral, with a slight male predominance.^3,4^ It may be associated with osseous and soft-tissue abnormalities involving the knee joints and the lower limbs such as femoral deficiency, hypoplasia of the lateral femoral condyle, shortening or bowing of the tibia, tarsal coalition, talipes equinovarus, missing lateral rays and cruciate ligament deficiency.^1^ We report an interesting case of fibular hemimelia diagnosed serendipitously, based on the characteristic radiographic and MRI findings in the knee joint and the lower limb.

## Case presentation

A 31-year-old male presented to the orthopaedic clinic of our hospital with symptoms of right knee pain and joint instability. He experienced a feeling of the knee giving away while walking. He had no history of trauma. There was no tenderness or swelling of the knee on physical examination and the range of motion was normal. The patient displayed positive Lachman and anterior drawer test. He also had congenital ankle and foot deformities. He was later sent for an MRI of the right knee to look for any internal derangement.

## Imaging findings

**Figure 1.**
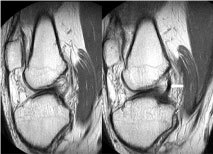
Sagittal proton density MRI. The midline image shows an absent anterior cruciate ligament while the more medial section shows a hypoplastic posterior cruciate ligament (arrow).

An MRI of the right knee was performed. No past history or imaging study was available at the time of reporting. The MRI study showed an absence of the anterior cruciate ligament (ACL) and a short posterior cruciate ligament (PCL) on the sagittal proton density (PD) images (Figure 1). Initially, the appearances were thought to be due to a chronic tear of the ACL and the PCL. However, the patient had no significant recent or past history of trauma, thus putting the radiologist in a dilemma.

**Figure 2.**
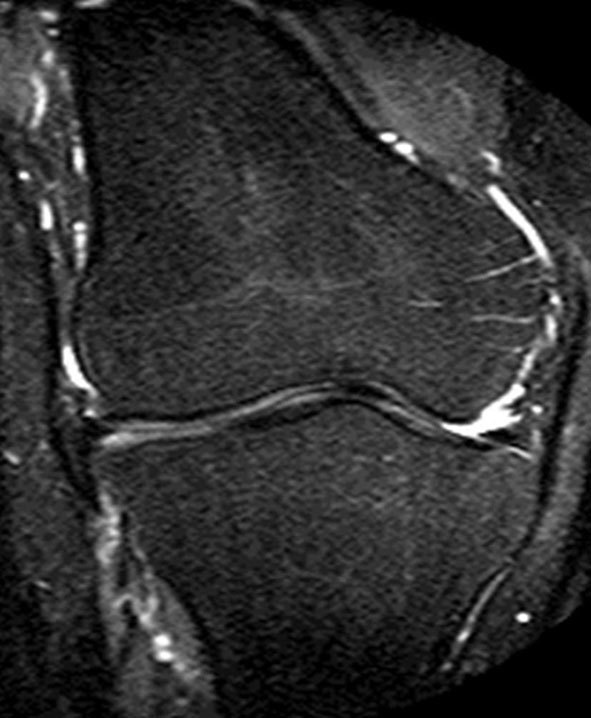
Coronal proton density fat-saturated MRI shows a hypoplastic tibial spine covered with cartilage.

**Figure 3.**
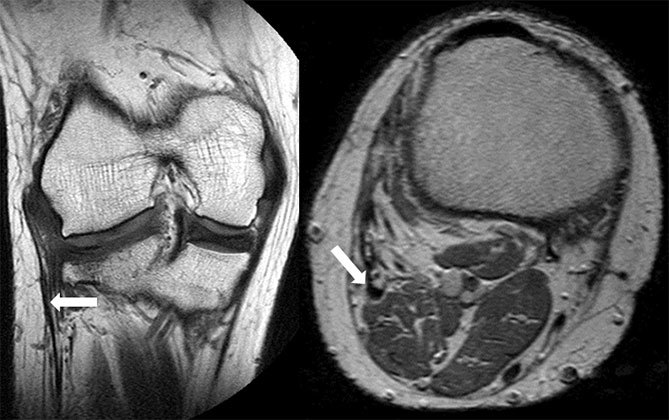
Coronal and axial proton density MRIs show an anomalous conjoint tendon (arrows) passing into the calf between the lateral gastrocnemius, popliteus and peroneal muscles.

**Figure 4.**
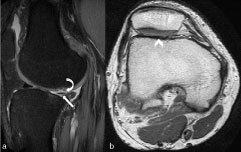
(a) Sagittal PD fat-saturated MRI shows a complex tear of the posterior horn of the lateral meniscus (straight arrow) with overlying cartilage injury (curved arrow). (b) Axial PD MRI shows a hypoplastic and flattened trochlear groove (arrowhead). PD, proton density.

On closer inspection, the tibial spine appeared hypoplastic on the coronal PD fat-saturated (FS) images, and was covered with cartilage (Figure 2). Furthermore, on the coronal and axial PD images, the proximal fibula could not be visualized, and the conjoint tendon of the lateral collateral ligament (LCL) and the biceps femoris continued distally into the soft tissues of the calf (Figure 3). These findings were considered secondary to some congenital abnormality. The additional findings were a complex tear of the posterior horn of the lateral meniscus with an adjacent cartilage defect and the flattening of the trochlear groove on the sagittal PDFS and axial PD images, respectively (Figure 4).

The patient was called back and questioned about any significant past medical or surgical history. He thereupon revealed having undergone multiple surgeries on his right limb during childhood for a congenital abnormality**, **including a limb lengthening procedure.

**Figure. 5.**
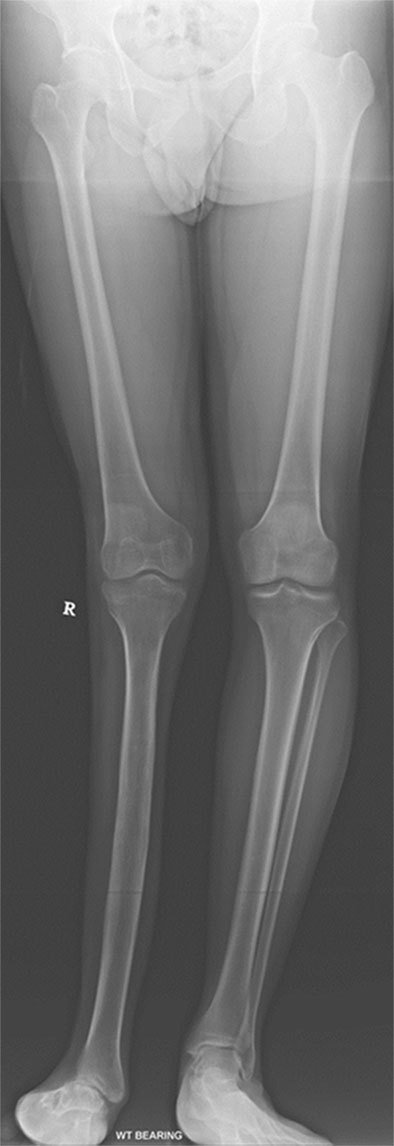
Full-length radiograph of the lower limb shows complete absence of the right fibula with genu valgus deformity of the right knee.

**Figure 6.**
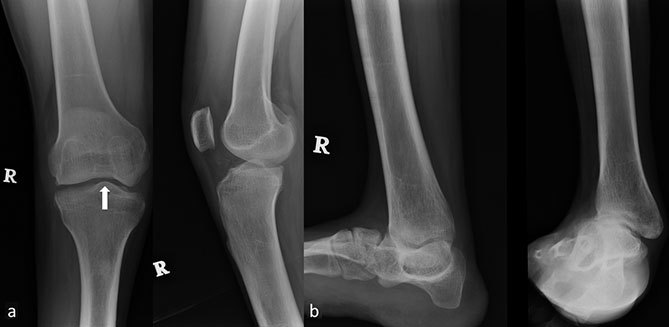
(a) Frontal and lateral radiographs of the right knee show a convex tibial eminence and a hypoplastic tibial spine (arrow). The lateral femoral condyle appears hypoplastic with anterior translation of the right tibia on lateral radiograph. (b) Frontal and lateral radiographs of the right ankle show severe ankle joint deformity.

Subsequently, the radiographs of the right knee and the ankle, as well as a full-length radiograph of both the lower limbs were taken. The full-length radiograph showed a complete absence of the right fibula with a genu valgus deformity (Figure 5). The frontal radiograph of the right knee showed a convex tibial eminence and a hypoplastic tibial spine. The lateral femoral condyle also appeared hypoplastic (Figure 6a). The lateral radiograph demonstrated an anterior translation of the right tibia (Figure 6a). The ankle radiograph revealed severe joint and foot deformities (Figure 6b).

## Differential diagnosis 

**Figure 7.**
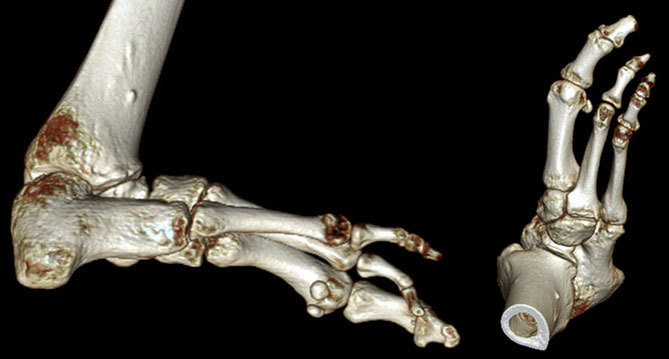
Three-dimensional reconstructed CT images of the right ankle and foot show club foot deformity, tarsal coalition and absent fourth and fifth rays.

The clinical history and imaging findings were consistent with a rare congenital abnormality known as fibular hemimelia. This condition is known for associated bone and soft-tissue abnormalities involving the knee, ankle and foot. A subsequent CT of the ankle and the foot showed absence of the lateral fourth and fifth rays, equinovarus deformity and tarsal coalition (Figure 7). On reviewing his previous records, it was found that the patient was a known case of fibular hemimelia and had undergone lengthening of the Achilles tendon, right foot centralization surgery and tibial lengthening procedure during childhood. He subsequently adapted well to his condition and was not just self-ambulant but was even able to play football socially. Surprisingly, in spite of known associations, he had never undergone an MRI of the knee before.

## Treatment

The case was discussed at the orthopaedic multidisciplinary meeting and a decision was taken to offer femoral osteotomy as the best course of treatment for the patient.

## Discussion

Fibular hemimelia is a rare congenital abnormality caused by longitudinal deficiency of the fibula. It represents a spectrum of deformities ranging from mild fibular hypoplasia to aplasia.^2^ Achterman and Kalamchi^5^ classified fibular hemimelia into two types based on the remaining fibula in the abnormal limb: type 1, where a portion of the fibula is present (1a: proximal fibular hypoplasia with an intact ankle mortise joint; 1b: 30–50% of the proximal fibula is absent with a dysplastic or absent ankle mortise joint) and type 2, where the fibula is completely absent or vestigial.^3^ Our patient corresponds to type 2 fibular hemimelia.

Fibular hemimelia, apart from an absent or hypoplastic fibula, is commonly associated with other skeletal abnormalities of the knees and the lower extremities.^3^ Imaging plays an important role in diagnosing and determining the disease severity, which then guides the management. Radiographs of the knee and the foot show either a hypoplastic or an aplastic fibula with other findings, including an absent or hypoplastic intercondylar notch, hypoplastic lateral femoral condyle and shallow trochlear groove. The proximal tibial epiphysis is commonly convex with absent or hypoplastic tibial spines. Other recognized features are tibial bowing and genu valgum deformity.^3^ A radiograph of the hip should also be taken to exclude proximal focal femoral deficiency.^2^ Our patient showed hypoplasia of the lateral femoral condyle, intercondylar dysplasia and hypoplasia of the tibial spine. The femur was otherwise normal with no proximal femoral deficiency.

Congenital absence of the cruciate ligaments is a very rare abnormality first described by Giorgi in 1956.^6^ It is usually associated with other congenital musculoskeletal disorders, most commonly longitudinal deficiencies of the lower limbs (e.g., congenital short femur and aplasia of the fibula or patella).^7^ In a study by Roux et al,^8^ the ACL was deficient in 95% and the PCL in 60% of patients with fibular hemimelia. The reported incidence of knee instability is between 3% and 50% in patients with fibular hemimelia with congenital absence of the ACL.^1^ An MRI of the knee is necessary in patients with fibular hemimelia to look for ACL deficiency and other internal derangements before deciding on a management protocol.^2^ The PCL is either absent or hypoplastic. The hypoplastic PCL is seen as a thin cord-like structure on the MRI and can be easily confused for the posterior joint capsule.^3^ If there is a history of trauma with a normal contralateral knee, the congenital absence of the ACL can be wrongly interpreted as an ACL tear.^7^

The congenital absence of the cruciate ligament was classified by Manner et al^9^ into three types: type 1, hypoplastic or absent ACL and a normal PCL; type 2, absent ACL with a hypoplastic PCL; and type 3, absence of both the cruciate ligaments. Our case falls in the type 2 category.

The posterolateral corner structures of the knee, such as the lateral collateral ligament (LCL), biceps femoris tendon and popliteofibular ligament, stabilize the lateral aspect of the knee and normally insert onto the fibular head.^3^ Therefore, in patients with fibular hemimelia, it is inevitable that there is some disruption of this normal anatomy. In our patient, an elongated conjoint tendon of the biceps femoris and the LCL descended into the soft tissues of the lateral aspect of the lower leg. Other findings that may be seen on the MRI are trochlear dysplasia, abnormal patellar tendon and a hypoplastic posterior horn of the lateral meniscus.^3^ Our case had a complex tear of the posterior horn of the lateral meniscus. Deficiency of the cruciate ligaments and abnormal posterolateral corner structures can result in joint subluxation, stress changes and early degenerative changes affecting the knee joint. Our patient showed early degenerative changes in the form of marginal osteophytes.

The treatment protocol for fibular hemimelia depends on the extent of leg-length discrepancy and foot function. A limb lengthening procedure is indicated in cases with mild-to-moderate foot deformity, with stable hip, knee and ankle.^10^ The use of Ilizarov’s method of limb lengthening has shown good results with preservation of the foot and significant limb lengthening.^11^ In cases with severe foot deformity, amputation (Syme’s and Boyd’s) is the preferred treatment.^10^ Changulani et al^11^ proposed that the number of rays in the foot can be used as an indicator of severity of foot deformity and a guide to deciding treatment. In their study, patients with three or more rays and with less severe limb deformities underwent the limb lengthening procedure. In contrast, children with less than three rays underwent Syme’s amputation.^11^

In conclusion, radiologists play a crucial role in determining the severity of fibular hemimelia, hence it is important for them to be aware of the typical features and associations of fibular hemimelia so as to be able to guide the orthopaedic management in these patients.

## Learning points

1. Fibular hemimelia is associated with various abnormalities in the knee joint and a radiologist needs to be aware of the typical features for guiding orthopaedic management in these patients.
2. Patients with fibular hemimelia should undergo an MRI of the knee joint owing to a consistent association with deficiencies of the cruciate ligaments and posterolateral corner structures.
3. The importance of a thorough past medical and surgical history in aiding the diagnosis of certain complex syndromes cannot be overemphasized.
